# Zinc homeostasis and neurodegenerative disorders

**DOI:** 10.3389/fnagi.2013.00033

**Published:** 2013-07-19

**Authors:** Bernadeta Szewczyk

**Affiliations:** Department of Neurobiology, Institute of Pharmacology Polish Academy of SciencesKrakow, Poland

**Keywords:** zinc, zinc transporters, metallothioneins, depression, aging, Alzheimer's disease, neurodegeneration

## Abstract

Zinc is an essential trace element, whose importance to the function of the central nervous system (CNS) is increasingly being appreciated. Alterations in zinc dyshomeostasis has been suggested as a key factor in the development of several neuropsychiatric disorders. In the CNS, zinc occurs in two forms: the first being tightly bound to proteins and, secondly, the free, cytoplasmic, or extracellular form found in presynaptic vesicles. Under normal conditions, zinc released from the synaptic vesicles modulates both ionotropic and metabotropic post-synaptic receptors. While under clinical conditions such as traumatic brain injury, stroke or epilepsy, the excess influx of zinc into neurons has been found to result in neurotoxicity and damage to postsynaptic neurons. On the other hand, a growing body of evidence suggests that a deficiency, rather than an excess, of zinc leads to an increased risk for the development of neurological disorders. Indeed, zinc deficiency has been shown to affect neurogenesis and increase neuronal apoptosis, which can lead to learning and memory deficits. Altered zinc homeostasis is also suggested as a risk factor for depression, Alzheimer's disease (AD), aging, and other neurodegenerative disorders. Under normal CNS physiology, homeostatic controls are put in place to avoid the accumulation of excess zinc or its deficiency. This cellular zinc homeostasis results from the actions of a coordinated regulation effected by different proteins involved in the uptake, excretion and intracellular storage/trafficking of zinc. These proteins include membranous transporters (ZnT and Zip) and metallothioneins (MT) which control intracellular zinc levels. Interestingly, alterations in ZnT and MT have been recently reported in both aging and AD. This paper provides an overview of both clinical and experimental evidence that implicates a dysfunction in zinc homeostasis in the pathophysiology of depression, AD, and aging.

## Introduction

Knowledge about zinc has rapidly evolved over the years with the last two decades having brought, interesting new insights about the role of zinc in molecular and cellular processes as well as health and disease. Zinc is one of the most prevalent trace elements in the human body. It is a key structural component of a great number of proteins, and a co-factor of more than 300 enzymes that regulate a variety of cellular processes and cellular signaling pathways essential for both brain and systemic physiology (Takeda, 2000). In the brain, zinc is also present in its free ionic form (Zn^2+^) within synaptic vesicles, mostly at the glutamatergic terminals (Frederickson et al., 2000; Paoletti et al., 2009; Sensi et al., 2011). Synaptically released zinc, during neuronal activity, affects the activity of N-methyl-D-aspartate (NMDA) and α-amino-3-hydroxyl-5-methyl-4-isoxazole-propionate (AMPA) glutamate receptors, GABA_A_ and glycine inotropic receptors (Smart et al., 2004). It has also been found to activate a specific metabotropic Zn2+-sensing receptor GPR39 (Besser et al., 2009). In physiological concentrations zinc exhibits neuroprotective activity, although high concentrations of zinc are neurotoxic (Choi et al., 1988; Perry et al., 1997; Cote et al., 2005; Plum et al., 2010). Therefore, an imbalance of zinc homeostasis will have complex implications in a number of brain processes then leading to the onset of chronic pathologies such as depression, schizophrenia, Alzheimer's disease (AD), Parkinson's disease, aging, or amyotrophic lateral sclerosis (ALS). Given the complex nature of zinc homeostasis in the brain, it is not surprising that several different groups of proteins are involved in managing its cellular levels. The first group consists of are membranous transporters (ZnTs) mediating the zinc efflux from cells or influx into cellular compartments or organelles (Huang and Tepaamorndech, 2013). The second group is members of the Zip family (zinc-regulated and iron-regulated transporter proteins) that promote zinc transport from the extracellular space or from intracellular vesicles to the cytoplasm (Cousins et al., 2006). So far, 10 members of the ZnT and 14 members of the ZIP protein families have been identified (Lichten and Cousins, 2009). The third group of these zinc homeostasis-regulating proteins is metallothioneins (MTs)—a group of low-molecular-weight metal-binding proteins that have a high affinity for zinc (Krezel et al., 2007). Four MT isoforms have been described so far; MT-I and MT-II are expressed in many tissues; MT-IV is exclusively expressed in some stratified squamous epithelia (Quaife et al., 1994) and MT-III. MT-III is a brain-specific member of the MTs protein family, found exclusively in neurons, and localized predominantly in neurons that sequester zinc in synaptic vesicles (Masters et al., 1994). MT-III mRNA has been found in the cortex, hippocampus, amygdala, and cerebellum (Masters et al., 1994). The role of MTs is to buffer cytoplasmic zinc following its influx into the cytoplasm, and so far it seems that temporary cellular zinc storage is the exclusive function of MTs (Krezel et al., 2007). MTs play a crucial protective role (due to their redox properties) in the presence of radiations, heavy toxic metals, lipid peroxidation, or reactive oxygen species (ROS) (Sato and Kondoh, 2002).

The understanding of the physiological functions of zinc transporters and MTs has grown dramatically during recent decade and their involvement in the pathogenesis of neurodegenerative diseases more apparent than previously. This review focuses on depression, AD, and age related pathologies, in which a specific role for zinc dyshomeostasis has been reported. Also, disease associated alterations in proteins responsible for zinc transport and zinc storage will be discussed.

## The role of zinc in modulating synaptic function

There are several important aspects associated with zinc depletion; supplementation and delivery of zinc to the brain. Experimentally, zinc deficiency is reached by partaking in a diet that contains 0.5 mgZn/kg–6 mgZn/kg for at least 2–4 weeks (Tamano et al., 2009; Mlyniec et al., 2012). Zinc overdoses can be obtained at 100 mgZn/kg–180 mgZn/kg (Yang et al., 2013). The most common way for assessing the zinc level is by measuring the serum or plasma zinc. Unfortunately, elevated or lowered serum zinc does not correspond with the elevated or lowered brain zinc total. This suggests that the brain zinc total is strictly controlled and may not be easily influenced by peripheral zinc level. The other problem is the lack of sensitive methods to measure alterations in the extra or intracellular zinc levels. Available data indicates that the hippocampus seems to be the most responsive both to the deficiency as well as an overdose of zinc (Takeda et al., 2005; Suh et al., 2009; Yang et al., 2013). Because the hippocampus is the region of the brain which plays a critical role in memory, learning and neurogenesis, the impact of zinc deficiency or zinc supplementation on these processes will be critical. Indeed, it was found that a zinc deficient diet, decreases the number of progenitor cells and immature neurons in the dentate gyrus (DG) in rodents and that reversal to a normal diet containing zinc restored a number of these cells (Gao et al., 2009; Suh et al., 2009). Reduced progenitor cells were also found after zinc chelator treatment and in ZnT3 KO mice (a lack of zinc in the synaptic vesicles) (Suh et al., 2009). A growing body of evidence indicates that dietary zinc deficiency influences hippocampal learning and memory in an age-dependent manner. It was found that a decrease in dietary zinc during early development produces an irreversible deficit of learning and memory, while zinc deficient induced impairments in young adult rats can be reversed by feeding them with an adequate diet (Takeda, 2000; Keller et al., 2001). Recent data published by Gao et al. (2009, 2011) showed that the zinc-deficiency induced hippocampal learning and memory impairments is in part due to the disruption of the calmodulin (CaM), CaM-dependent protein kinase II (CaMKII), and cAMP-responsive element binding protein (CREB) signaling pathway. As was mentioned above zinc was found to modulate neural transmission through the GPR39 Zn2+-sensing receptor. Recent studies showed a significant reduction in the GPR39 protein level in the frontal cortex in mice receiving the zinc deficient diet (Mlyniec et al., 2013). This study provides evidence that the GPR39 Zn2+-sensing receptor may be involved in the pathomechanism of depression. This hypothesis was further supported by data indicating the up-regulation of the GPR39 receptor after chronic antidepressant treatment (Mlyniec and Nowak, 2013).

The other mechanism by which zinc can modulate synaptic functions is the transactivation of the tropomyosin-related kinase B (TrkB) receptor and activation of brain-derived neurotrophic factor BDNF signaling in a neurotrophic—independent manner (Huang et al., 2008). Zinc can affect BDNF signaling also by promoting the maturation of pro-BDNF to BDNF throughout the activation of metalloproteinases (MMPs) (Hwang et al., 2005).

Zinc also appears to have an effect of oxidative stress. It was found that both high and extremely low concentrations of zinc are associated with increased oxidative and nitrosative stress [by increasing the expression of neuronal nitric oxide synthase (nNOS) and NADPH oxidase] (Noh and Koh, 2000; Aimo et al., 2010), however, intermediate concentrations was found to be neuroprotective (Aimo et al., 2010). This demonstrates once again the importance of zinc homeostasis in normal brain function. Although, the effect of zinc deficiency on the brain zinc homeostasis and learning and memory has been well studied, the effect of a zinc overdose on these processes is poorly described and the data are rather conflicting. First, the effect of zinc supplementation on learning and memory impairments is dose dependent. Generally, zinc supplementation in a low dosage seems to improve the performance of animals in spatial memory tasks (Piechal et al., 2012) or the contextual discrimination task (Yang et al., 2013). However, memory deficits in rats after low dose of zinc supplementation were also observed (Flinn et al., 2005; Railey et al., 2010). Interestingly Yang et al. (2013) reported that zinc supplementation in high doses induce a dramatic decrease in hippocampal zinc levels, especially in the CA3 and DG, and impaired learning and memory due to a decreased availability of synaptic zinc and BDNF deficits.

## Zinc and depression

Depression is a common mental disorder associated with functional impairment, significant disability, morbidity and mortality. Despite the extensive research that has so far been carried out on depression, its pathophysiology is still poorly understood. One of the many hypotheses proposed for depressive disorder indicates that depression is characterized by an enhanced neurodegeneration and decreased neurogenesis (Maes et al., 2009). On the other hand, there is increasing evidence linking depression or depression-related changes in brain function or cognitive performance to zinc ion availability.

### Zinc levels in depression (Table 1)

Clinical studies demonstrate significantly lower serum zinc levels in patients suffering from major depression or unipolar depression than that in non-depressed patients (McLoughlin and Hodge, 1990; Maes et al., 1994, 1997; Nowak et al., 1999). In some patients, a negative correlation between the serum zinc level and severity of depression was found (Maes et al., 1994; Nowak et al., 1999). A lower serum zinc level was also found to accompany antepartum and postpartum depression. In this study the level of zinc was also negatively correlated with the severity of depressive symptoms (Wojcik et al., 2006). Low serum zinc levels have also been noted in depressed patients with end-stage renal disease undergoing hemodialysis (Roozbeh et al., 2011). Moreover, treatment-resistant depressed patients have been shown to exhibit much lower serum zinc concentrations than their non-treatment resistant depressed counterparts (Siwek et al., 2010). Thus far, only two studies have reported no differences in the zinc level between depressed and non-depressed patients (Narang et al., 1991; Irmisch et al., 2010). The paper published by Irmisch et al. (2010), however indicated that zinc concentrations might differ dependent on comorbid disorders and severity of depression. Similarly, Narang et al. (1991) reported no significant difference between control and depressed patients, however, they found that the values were significantly higher in recovered patients compared to patients with depression. Although these results do not confirm the general hypothesis of a lack of zinc in depressive disorders, however favor the existence of correlation between severity of depression or status of patients and zinc concentration.

**Table 1:** **Summary of the main clinical and preclinical findings supporting the involvement of zinc in depression**.

**Serum/plasma zinc status—human data**	**References**
↓ Major depressed patients; negative correlation between the serum zinc and severity of depression	Maes et al., 1994; Nowak et al., 1999
↓ Depressed patients vs. control	Siwek et al., 2010
↓ Patients with affective disorders	McLoughlin and Hodge, 1990
↓ Women with antepartum and postpartum depressive symptoms	Wojcik et al., 2006
↓ Depressed patients with end-stage renal disease undergoing hemodialysis	Roozbeh et al., 2011
↔ Depressed patients; zinc concentrations differ dependent on comorbid disorders and severity of depression	Irmisch et al., 2010
↔ Depressed patients; significantly higher zinc level in recovered patients compared to patients with depression	Narang et al., 1991
**Effect of zinc deficiency—human study**	
Correlation between dietary zinc intake and the serum zinc concentrations; the inverse correlation between serum zinc levels and depression scales	Amani et al., 2010
Zinc intake moderates the association between stress and depressive symptoms	Roy et al., 2010
**Effect of zinc deficiency—animal study**	
↑ Immobility time in FST in rats	Tassabehji et al., 2008; Tamano et al., 2009; Watanabe et al., 2010
↑ Immobility time in FST in mice	Whittle et al., 2009; Mlyniec et al., 2012
↑ Immobility time in TST in mice	Whittle et al., 2009; Mlyniec and Nowak, 2012
↓ Saccharin preference in rat	Tassabehji et al., 2008
**Effect of zinc treatment/supplementation in depression—clinical trials**	
Zinc supplementation (25 mg/12 weeks) significantly reduced scores in HDRS and BDI when compared with placebo treatment	Nowak et al., 2003a
Zinc supplementation (25 mg/12 weeks) augments the efficacy and speed of onset of therapeutic response to imipramine treatment, particularly in patients previously non-responsive to antidepressant pharmacotherapies	Siwek et al., 2009
Zinc supplementation (25 mg/12 weeks) significantly reduced HDRS compared to placebo	Ranjbar et al., 2013
Women who took multivitamins and zinc (7 mg/10 weeks) showed a significant reduction in anger-hostility score and depression-dejection score in the Profile of Moods State (POMS)	Sawada and Yokoi, 2010
Zinc deficiency changes the brain function but zinc and macronutrient treatment improves altered brain functions	Sandstead, 2012
Zinc supplementation (10 mg/6 months) did not induce differences in mental health outcomes between zinc and placebo groups, however, increases in serum zinc concentrations were associated with decreases in internalizing symptoms (depression and anxiety)	DiGirolamo et al., 2010
No effect of zinc supplementation on the improvement of depressive symptoms	Nguyen et al., 2009
**Effect of zinc treatment—animal study**	
↓ In immobility time in both FST and TST	Kroczka et al., 2000, 2001; Nowak et al., 2003b; Rosa et al., 2003; Cunha et al., 2008; Franco et al., 2008
↓ Reduction in the number of trials in the passive-avoidance test in OB model; ↓ OB- induced hyperactivity in open field test in OB model	Nowak et al., 2003b
Zinc reversed the CMS-induced reduction in the consumption of sucrose	Sowa-Kucma et al., 2008
Zinc prevented deficits in the fighting behavior in CUS model	Cieslik et al., 2007
Zinc intensifies the effects of standard antidepressants in FST, TST, and CUS	Szewczyk et al., 2002, 2009; Rosa et al., 2003; Cieslik et al., 2007; Cunha et al., 2008

### Zinc deficiency and depression

There is a paucity of clinical studies that have examined the relationship between dietary zinc intake and depressive symptoms (Table 1). One study, carried out by Amani et al. (2010) showed that both daily zinc intake and the serum zinc levels in young depressed women were about two thirds of that observed in healthy volunteers. Moreover, an inverse correlation was found between serum zinc concentrations and depression scale scores. In another study, conducted among a group of pregnant women, the relationship between dietary zinc intake, psychosocial stress and sociodemographic factors and depression was examined. Analysis of the results showed that lower zinc intake, higher stress and social disadvantage were associated with the occurrence of depressive symptoms, which were in turn attenuated by higher zinc intake (Roy et al., 2010). Data coming from animal studies further support the hypothesis that a deficiency in zinc can lead to the induction of depressive behavioral symptoms (Table 1). Studies have shown that zinc-deficient mice exhibit an increased immobility time in the forced swim test (FST) and tail suspension test (TST) (Whittle et al., 2009; Mlyniec and Nowak, 2012; Mlyniec et al., 2012). Pro-depressive-like behavior (increased immobility in the FST or anhedonia) was also found in rats subjected to zinc-deprivation (Tassabehji et al., 2008; Tamano et al., 2009; Watanabe et al., 2010)

### Zinc treatment/supplementation in depression

Some clinical studies have shown the beneficial effect of zinc supplementation in the treatment of depression (Table 1). One such study by Nowak et al. (2003a), was conducted in depressed patients, treated with tricyclic antidepressants and selective serotonin reuptake inhibitors supplemented with zinc or a placebo. Analysis of the Hamilton Depression Rating Scale (HDRS) and Beck Depression Inventory (BDI) scores revealed that patients who received the zinc supplementation of antidepressant treatment displayed much lower scores than patients treated with placebos and antidepressants. A beneficial effect of zinc as an adjunct agent was also found in treatment-resistant patients (Siwek et al., 2009). In this placebo-controlled, double blind study patients were randomized into two groups: the first were treated with imipramine and received one daily placebo and the second were treated with imipramine supplemented with zinc. It was found that zinc supplementation significantly reduced the depression scores [measured by Clinical Global Impression (CGI); Montgomery-Asberg Depression Rating Scale (MADRS); BDI and HDRS] and facilitated the effect of the treatment in antidepressant treatment resistant patients. No significant differences in the CGI, MADRS, BDI, and HDRS scores were demonstrated between zinc and placebo- supplemented antidepressant treatment non-resistant patients. The benefit of zinc supplementation in patients with major depression has been recently reported by Ranjbar et al. (2013). This randomized, double-blind, placebo-controlled trial is the next clinical study indicated that zinc supplementation in conjunction with antidepressants might be beneficial for reducing depressive symptoms.

The other study published by Sawada and Yokoi (2010) showed that young women taking multivitamins and zinc supplements exhibited a significant reduction in depression and anxiety symptoms than women taking only multivitamins. In 2012, Sandstead published the results from six randomized controlled comparative treatment experiments in Chinese and Mexican-American low-income children, aged 6–9 years; middle-income US premenopausal women; middle income US adolescents and middle- income US men, illustrating that subclinical zinc deficiency changes the brain function and that zinc and micronutrient treatment improves altered brain functions (Sandstead, 2012). Two studies have so far shown no effect of zinc supplementation on the improvement of depressive symptoms (Nguyen et al., 2009; DiGirolamo et al., 2010). However, these studies differ significantly from that previously described with respect to both the patients and the length and quality of applications. The first study by DiGirolamo et al. (2010) examined the effect of six months of zinc supplementation on the mental health of school-age children. The second study, investigated the impact of combinations of micronutrient supplements on symptoms of depression rather than effect of zinc supplementation as a stand-alone. Because of these methodological limitations in existing studies, further well-designed, adequately powered research is required.

The beneficial effects of zinc treatment have been also reported in preclinical studies (Table 1). Zinc administration induced an antidepressant-like effect (reduction in immobility time) in both the FST and TST (Kroczka et al., 2000, 2001; Nowak et al., 2003b; Rosa et al., 2003; Cunha et al., 2008; Franco et al., 2008). Zinc was also active in different models of depression. In the olfactory bulbectomy (OB) a reduction in the number of trials in the passive-avoidance test and a decreased OB-induced hyperactivity in rats after zinc treatment was observed (Nowak et al., 2003b). While in the chronic mild stress (CMS) model of depression; zinc reversed the CMS-induced reduction in the consumption of sucrose in rats (Sowa-Kucma et al., 2008). In chronic unpredictable stress (CUS) in turn, zinc treatment prevented deficits in the fighting behavior of chronically stressed rats (Cieslik et al., 2007). Moreover, zinc has been found to intensify the effects of standard antidepressants (IMI, fluoxetine, paroxetine, bupropion, or citalopram) in the FST, the TST, and CUS (Szewczyk et al., 2002, 2009; Rosa et al., 2003; Cieslik et al., 2007; Cunha et al., 2008).

Presented above data strongly indicated the importance of zinc deficiency in human depression and indicated the benefit of zinc supplementation in both the efficacy and the speed of the therapeutic response to antidepressants treatment. Thus, the understanding of the mechanisms involved in the antidepressant activity of zinc might contribute to the development of a new therapeutic strategy for the treatment of depression or depression-related diseases. Published so far data points out that the modulation of glutamatergic neurotransmission (via the NMDA or AMPA glutamate receptors), serotonergic transmission (especially via the 5-HT1A receptor) and regulation of BDNF level seems to be the most important interactions involved in the antidepressant-like activity of zinc (Nowak et al., 2004; Sowa-Kucma et al., 2008; Cichy et al., 2009; Szewczyk et al., 2009, 2010).

## Zinc and alzheimer's disease

AD is a chronic neurodegenerative disorder and the most common cause of dementia. It is estimated that AD represents 60–80% of all dementia cases (Daviglus et al., 2010). The clinical features of AD vary from stable performance and cognitive health with only a gradual decline in the short-term memory to a serious state of cognitive impairment and into different forms of dementia (deterioration of memory, learning, orientation) (Daviglus et al., 2010). On the other hand the pathological features of AD is the accumulation of β-amyloid (Aβ) and the aggregation of Aβ is suggested as the cause of neurodegeneration observed in AD (Small and Cappai, 2006).

Although the key role of Aβ in the pathogenesis of AD is strongly established now, the mechanism by which Aβ induces toxicity or the causes and factors associated with the risk or progression of AD is still poorly understood. One of the several hypotheses proposed for the pathophysiology of AD is the trace elements hypothesis, with zinc taking the center stage. Zinc was first described as a possible factor leading to dementia by Burnet (1981) and, since then, the knowledge base regarding the role of zinc in the pathogenesis and therapy of AD has evolved rapidly.

### Zinc levels in AD

Serum, cerebrospinal fluid (CSF) and brain zinc levels have been investigated in patients diagnosed with AD (Table 2). Several of these studies investigating serum zinc levels have shown either divergent data with no differences (Shore et al., 1984; Haines et al., 1991), a significant decrease (Jeandel et al., 1989; Baum et al., 2010; Brewer et al., 2010; Vural et al., 2010) or a significant increase (Gonzalez et al., 1999; Rulon et al., 2000) when compared to matched controls. The main problem associated with these clinical studies is that different methodologies and different selections of patients were used meaning that the end result could account for the various divergent data obtained in the studies. Studies looking at CSF zinc levels also showed some discrepancies. For instance, Hershey et al. (1983) and Sahu et al. (1988) found no differences in CSF levels of zinc in patients with dementia of the Alzheimer type relative to a matched group of healthy controls. In contrast, Molina et al. (1998) found a significant decrease in CSF zinc levels in AD patients than the control subjects.

**Table 2:** **Summary of the main clinical and preclinical findings supporting the involvement of zinc in AD**.

**Serum/CSF/brain zinc status—human data**	**References**
↔ Serum zinc level in patients with and without cognitive impairment in the community	Haines et al., 1991
↔ Serum and hair zinc concentration in patients with AD	Shore et al., 1984
↑ Serum zinc level in AD epsilon 4 apoE allele carriers	Gonzalez et al., 1999
↑ Zinc serum in AD subjects compared with age-matched control subjects-postmortem study	Rulon et al., 2000
↓ Serum zinc level in AD patients	Baum et al., 2010
↓ Blood zinc in patients with AD than in controls	Brewer et al., 2010
↓ Serum zinc level in patients with senile dementia of the Alzheimer type (SDAT) when compared to control subjects	Jeandel et al., 1989
↓ Plasma zinc level in patients with AD compared with controls	Vural et al., 2010
↔ In CSF zinc level in patients with dementia of the Alzheimer type	Hershey et al., 1983; Sahu et al., 1988
↓ CSF zinc levels in AD patients as compared with controls	Molina et al., 1998
↓ Hippocampal zinc concentration in patients with AD—postmortem study	Corrigan et al., 1993
↓ Zinc level in both hemispheres of the superior frontal gyrus, the superior parietal gyrus, the medial temporal gyrus, the hippocampus and the thalamus in the AD patients—postmortem study	Panayi et al., 2002
↑ Zinc level in hippocampus and amygdala in AD patients—postmortem study	Danscher et al., 1997
↑ Tissue zinc in the AD-affected cortex compared with the control group	Religa et al., 2006
↑ Zinc in olfactory regions of AD patients as compared to control subjects	Samudralwar et al., 1995
↑ Zinc in amygdala and hippocampus in AD patients as compared to controls	Thompson et al., 1988
**Zinc dyshomeostasis as a new therapeutic target in AD—animal study**	
Administration of DP-109 (the lipophilic metal chelator) reduced the aggregation of Aβ protein and deposition of amyloid plaques in aged female hAbetaPP-transgenic Tg2576 mice, compared to animals receiving vehicle treatment	Lee et al., 2004
Clioqunol (metal chelator) reduced zinc accumulation in the neuritic plaques and inhibit amyloidogenic AβPP processing in the AβPP/PS1 mouse brain	Wang et al., 2012
Carnosine supplementation in 3 × Tg-AD mice promotes a strong reduction in the hippocampal intraneuronal accumulation of Aβ and completely rescues AD and aging-related mitochondrial dysfunctions	Corona et al., 2011
Selective intracellular release of zinc ions from bis(thiosemicarbazonato) complexes reduces levels of Alzheimer disease amyloid-beta peptide	Donnelly et al., 2008
Presenilins are important for cellular zinc turnover and has the potential to indirectly impact β-amyloid aggregation through metal ion clearance	Greenough et al., 2011
Zinc supplementation delays hippocampal-dependent memory deficits and reduces both Aβ and tau pathology in the hippocampus	Corona et al., 2010
**Zinc dyshomeostasis as a new therapeutic target in AD—human study**	
PBT2 (copper/zinc ionophore) lowered CSF levels of Aβ and significantly improved cognition in AD patients	Lannfelt et al., 2008; Faux et al., 2010

Studies investigating zinc content in brain tissue suggests that an alteration in the zinc level seems to be fraction/region specific. Studies involving whole tissue samples have shown no differences in brain zinc levels between AD and the control subjects. Although, some alterations in the brain zinc levels were found when tissue was sub-fractionated (a decrease in nuclear but not in mitochondrial or microsomal fractions) or when different brain regions were analyzed separately (Wenstrup et al., 1990). Indeed, decreased zinc levels have been found in the neocortex, medial temporal gyrus, thalamus, and hippocampus (Corrigan et al., 1993; Panayi et al., 2002), whilst increased levels were found in the amygdala, hippocampus, cerebellum, olfactory areas and superior temporal gyrus (Thompson et al., 1988; Samudralwar et al., 1995; Danscher et al., 1997; Religa et al., 2006). The above mentioned data, even though inconsistent, strongly support the hypothesis that a deregulated zinc homeostasis is involved in the pathophysiology of AD.

### Role of zinc in AD—possible molecular mechanisms

Amongst all the multiple roles of zinc in the pathogenesis of AD, the most widely studied is the involvement of zinc in the accumulation of Aβ. Post-mortem studies using different imaging techniques for zinc analysis have demonstrated significant increases in zinc levels in neuropil and plaques present in the brain of AD patients when compared to normal age-matched controls (Lovell et al., 1998; Suh et al., 2000; Dong et al., 2003; Miller et al., 2006). On the other hand, lack of synaptic zinc prevents Aβ deposition (Lee et al., 2002).

Aβ is the product of proteolytic cleavage from the amyloid precursor protein (APP) by the enzyme known as β-secretase or β-site APP cleaving enzyme-1 (BACE-1) (Masters et al., 1985). Several pathways for the involvement of zinc in APP processing or Aβ aggregation has been suggested. It was found that APP synthesis is regulated by zinc-containing transcription factors NF-_κ_B and sp1 (Grilli et al., 1996). Zinc is also involved in processing of APP protein (Lee et al., 2009). The processing of APP relies on a number of activities by enzymes secretases (α-,β-, and γ-). The predominant route by which APP is processed in the brain is cleavage by the α-secretase, within the Aβ region, producing sAPP (soluble amyloid precursor peptide) (Ling et al., 2003). Further processing by the β-secretase and γ-secretase leads to the formation of Aβ peptide (Wilquet and De, 2004). It was found that APP contains a specific zinc binding site localized in the cysteine-rich region of the APP ectodomain (spanning the α-secretase position) (Bush et al., 1994b) and it is suggested that changes in the intracellular concentration of zinc may influence the relative activities of APP secretases (Bush et al., 1994a,b). However, is worth mentioning that zinc is clearly not the only factor influencing APP processing and its role has not been fully determined.

Recent evidence suggests that oxidative stress is an additional factor contributing to the progression of AD (Butterfield et al., 2001; Jomova et al., 2010) and that ROS or exogenous oxidants are able to promote a harmful zinc release from MTs (Aizenman et al., 2000; Bossy-Wetzel et al., 2004). Zinc accumulation can in turn, induce mitochondrial dysfunction and further ROS generation (Sensi et al., 2003). Results presented by Sensi et al. (2008) indicate that such ROS-dependent intraneuronal zinc rises are particularly high in AD neurons expressing mutant APP, presenilin-1 (PS-1) and tau (Sensi et al., 2008).

### Zinc dyshomeostasis as a new therapeutic target in AD

Considering the fact that zinc contributes to the aggregation of the Aβ protein and deposition of amyloid plaques in AD, research has been focused on the use of metal complexation ability as therapeutic agents in AD (Table 2). Indeed, the metal chelator, clioquinol (CQ) and zinc modulator—DP-109 were found to significantly decrease the formation of amyloid plaques in the brains of APP/PS1 double transgenic mice and aged female hAbetaPP-transgenic Tg2576 mice, respectively (Lee et al., 2004; Wang et al., 2012). Faux et al. (2010) describes a successful phase 2 clinical trial of the quinoline derivative, PBT2 in AD (Faux et al., 2010). This randomized, placebo controlled trial found that this metal-protein attenuating compound (MPAC) that affects the Cu2(+)-mediated and Zn2(+)-mediated toxic oligomerization of Abeta seen in AD significantly lowered the CSF levels of Aβ and significantly improved cognition in AD patients (Lannfelt et al., 2008; Faux et al., 2010). The other possible therapeutic compound for AD, suggested recently is carnosine—a peptide with cooper/zinc chelating properties (Trombley et al., 1998). Corona et al. (2011) found that dietary supplementation of carnosine reduces hippocampal intraneuronal accumulation of Aβ and rescues mitochondrial dysfunctions in triple-transgenic AD mice (3 × Tg-AD) but does not affect the development of the tau pathology and only slightly reduces cognitive deficits (Corona et al., 2011). Furthermore, the paper of Donnelly et al. (2008) demonstrated the beneficial effect of the selective intracellular delivery of zinc using bis(thiosemicarbazonato) complexes in the reduction of the extracellular levels of Aβ and suggested the role of these metal-loaded compounds as potential therapeutic agents for AD (Donnelly et al., 2008). In turn Greenough et al. (2011) reported that presenilin, which mediates the proteolytic cleavage of the β-amyloid precursor protein to release β-amyloid, is important for cellular cooper/zinc turnover and has the potential to indirectly impact on amyloid aggregation through zinc ion clearance (Greenough et al., 2011).

Interestingly, a delay in hippocampal-dependent memory deficits and reduction of both the Aβ and tau pathology in the hippocampus in 3 × Tg-AD mice was also observed after zinc supplementation (Corona et al., 2010). This study also indicated the involvement of the BDNF-tyrosine kinase type B (TrkB) receptor pathway in the mechanism of the beneficial effect of zinc supplementation in this AD model (Corona et al., 2010).

All of these data further emphasizes the integral role of zinc in the mechanism of AD and support the hypothesis that restoring zinc homeostasis might be beneficial in the treatment of AD, although it also indicated the complex interactions between AD and zinc.

### Role of metallothioneins and zinc transporters in AD

**Figure 1:**
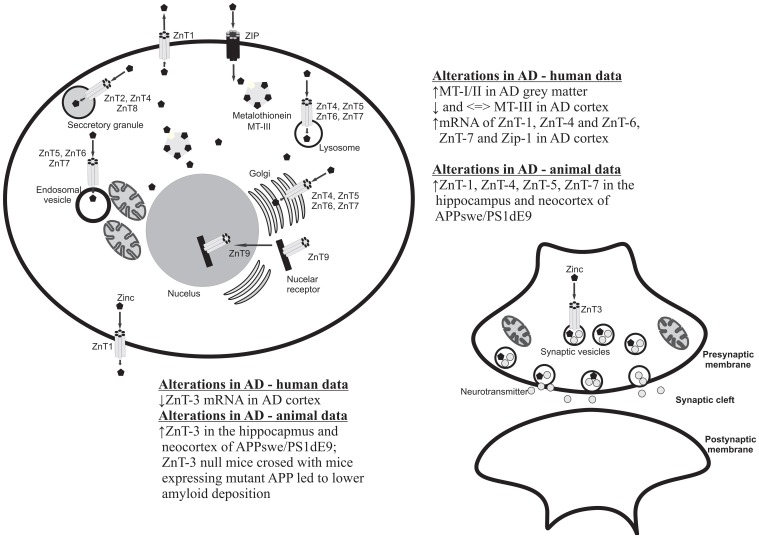
**Cellular localization of zinc transporters and metallothioneins and summary of the main clinical and preclinical findings supporting the involvement of these proteins in AD**.

There are several proteins/pathways that interact with zinc and that are also relevant to AD (Figure 1). One of these is MT. As noted earlier, MTs are zinc- binding proteins involved in the regulation of the transport, storage and transfer of zinc to various enzymes and transcription factors (Liuzzi and Cousins, 2004; DiGirolamo et al., 2010). The involvement of MTs in the regulation of zinc homeostasis makes it important in the context of the Zinc hypothesis of AD. Indeed, there are a number of studies that have reported increases, decreases and no change in MT isoforms in the brain. The study published by Adlard et al. (1998) showed a significant increase in MT I/II in the gray matter of preclinical AD cases when compared to non-AD cases. The authors suggested that the increase in MT I/II might be associated with the initial stages of AD processes due to the oxidative stress or alterations in the metabolism of heavy metals (Adlard et al., 1998). MT-III, also known as the growth inhibitory factor (GIF) was found to be down-regulated in the AD cortex (Uchida et al., 1991; Tsuji et al., 1992; Cuajungco and Lees, 1997; Yu et al., 2001), although no changes in the MT-III level in AD was also observed (Erickson et al., 1994; Amoureux et al., 1997). These discrepancies across different studies may result from the stage of the disease or cellular zinc status. Another important group of proteins involved in the homeostasis of zinc and the pathogenesis of AD are zinc transporter (ZnTs) proteins. Zhang et al. (2008) showed that six ZnTs such as: 1–7 are extensively present in the Aβ, being therefore positive plaques in the cortex of human AD brains. Recent studies showed alterations in levels of ZnTs proteins in the brain of subjects diagnosed with the preclinical stage of AD (PCAD), mild cognitive impairment (MCI), early (EAD), and the late (LAD) stage of AD when compared to the control subjects (Lovell et al., 2005, 2006; Smith et al., 2006; Lovell, 2009; Lyubartseva et al., 2010; Lyubartseva and Lovell, 2012). Human postmortem brain tissue from Braak-staged individuals with AD displayed a reduced expression of ZnT-3 mRNA (Beyer et al., 2009) and increased mRNA levels of the other more established zinc transporters, such as LIV1, ZIP1, ZnT1, ZnT6 in the AD cortex (Beyer et al., 2012). Also animal studies have linked dyshomeostasis in the brain zinc level to the pathogenesis and progression of AD. Lee et al. (2002) using a ZnT-3 null mice crossed with mice expressing mutant APP showed that the absence of synaptic zinc reduces the plaque load and increases the ratio of soluble/insoluble Aβ species. As such, this data suggested that synaptic zinc plays a key role in Aβ aggregation and plaque accumulation. Other studies also reported that with aging, female mice exhibit higher levels of synaptic, insoluble Aβ and plaques than males and that these sex differences disappeared in ZnT-3 knockout mice, correlating with the well described age-adjusted increase incidence for AD in females rather than males (Katzman et al., 1989). Recent studies published by Zhang et al. (2010) showed significant increases of ZnT-1, ZnT-3, ZnT-4, ZnT-6, and ZnT-7 in the hippocampus and neocortex of APPswe/PS1dE9 transgenic mice which corresponding to a form of early onset AD. Lang et al. (2012) in turn demonstrated that over-expression of Drosophila homolog of human Zip1 results in zinc accumulation in Aβ42–expressing fly brains and that inhibition of Zip1 expression induces a reduction of Aβ42 fibril deposits and improves cognition (Lang et al., 2012).

## Zinc in brain aging

Aging is an inevitable process associated with progressive pathological features such as: oxidative stress, altered cell metabolism, damaged of nucleic acid, or deposition of abnormal forms of proteins. In the brain aging is characterized by neuronal loss, cognitive impairment, and susceptibility to neurological disorders (Mocchegiani et al., 2005).

Recent progress in studies involving age related processes provide evidence that changes occurring in the brain during aging are related to zinc homeostasis and that zinc deficiency is a common cause of morbidity among the elderly (Mocchegiani et al., 2005). In aging, zinc deficiency is usually the result of an inadequate zinc dietary intake. It has been reported that only 40% of elderly people have a sufficient intake of zinc (Andriollo-Sanchez et al., 2005; Mocchegiani et al., 2008). Studies comparing old and young mice fed with low dietary zinc indicated that zinc is an important nutritional factor for a proper inflammatory/immune response (Kelly et al., 1996). Accordingly, zinc has anti-inflammatory properties and a low zinc status is associated with increased susceptibility to infection plus intracellular zinc has been found to play a key role in signaling in immune cells (Haase and Rink, 2009; Hasan et al., 2012). On the other hand aging is characterized by the progressive dysregulation of immune responses. Therefore, zinc has been suggested as a good factor in providing the remodeling of some age-associated changes and also as leading to healthy ageing through the reduction of inflammation (Kahmann et al., 2008). The study by Wong et al. (2013) suggests that age-related epigenetic dysregulation in ZnT expression may change cellular zinc levels and increase inflammation with age. They found that reduced Zip6 expression enhanced proinflammatory responses and that this age-induced Zip6 dysregulation correlated with an increased Zip6 promoter methylation. Interestingly, dietary supplementation reduced aged-associated inflammation (Wong et al., 2013). The other mechanism linking age, zinc and inflammation is associated with MTs. It was found that ageing is associated with a higher MT expression and consequently, low availability of intracellular zinc for normal immune responses. On the other hand, the supplementation of zinc in aging improves immune function and leads to decreased mortality from infections (Mocchegiani et al., 2010). In another study, Mocchegiani et al. (2011) showed evidence that zinc deficiency and an altered immune response is more evident in people with a polymorphism in IL-6 and metal-response element binding transcription factor-1 (MT1A) and that these individuals will benefit more from zinc supplementation.

## Conclusions

From the foregoing results, it is obvious that zinc homeostasis may play a major role in the initiation and propagation of the pathological features of psychiatric and neurodegenerative disorders. However, more studies are needed to explain the exact mechanisms linking zinc and processes related to these diseases.

First, since zinc deficiency is prevalent in patients with psychiatric and neurodegenerative disorders, the appropriate preventive measures should be considered especially in the elderly. Conversely, even if the beneficial effects of zinc supplementation were reported either in treatment or in the prevention of depressive or aging symptoms, zinc supplement users should be overly cautious and avoid overdosing.

Some of the studies presented above suggest that zinc can be useful not only by itself but in combination with other drugs used in treatment. Other important aspects in the context of zinc and treatment of patients are metal chelating drugs, for which the positive effect was particularly emphasized in AD. The weakness in most of these drugs, however, are the side effects caused by the chelation of other important divalent metal ions in the brain. Chelation should thus be used only when the brain zinc level is expected to have neurotoxic effects.

Recently, the zinc-homeostasis regulating proteins such as transporters and MTs have been gaining more prominence in related literature indicating they may be very important players in the pathophysiology of neurodegenerative disorders. Therefore, more studies are needed to fully understand the influence of peripheral zinc deficiency or an overdose on these proteins.

### Conflict of interest statement

The author declares that the research was conducted in the absence of any commercial or financial relationships that could be construed as a potential conflict of interest.

## References

[B1] Adlard P. A. West A. K. Vickers J. C. (1998). Increased density of metallothionein I/II-immunopositive cortical glial cells in the early stages of Alzheimer's disease. Neurobiol. Dis. 5, 349–356 10.1006/nbdi.1998.0203 10069577

[B2] Aimo L. Cherr G. N. Oteiza P. I. (2010). Low extracellular zinc increases neuronal oxidant production through nadph oxidase and nitric oxide synthase activation. Free Radic. Biol. Med. 48, 1577–1587 10.1016/j.freeradbiomed.2010.02.040 20211250 PMC3506424

[B3] Aizenman E. Stout A. K. Hartnett K. A. Dineley K. E. McLaughlin B. Reynolds I. J. (2000). Induction of neuronal apoptosis by thiol oxidation: putative role of intracellular zinc release. J. Neurochem. 75, 1878–1888 10.1046/j.1471-4159.2000.0751878.x 11032877

[B4] Amani R. Saeidi S. Nazari Z. Nematpour S. (2010). Correlation between dietary zinc intakes and its serum levels with depression scales in young female students. Biol. Trace Elem. Res. 137, 150–158 10.1007/s12011-009-8572-x 20013161

[B5] Amoureux M. C. Van G. D. Herrero M. T. Dom R. Colpaert F. C. Pauwels P. J. (1997). Regulation of metallothionein-III (GIF) mRNA in the brain of patients with Alzheimer disease is not impaired. Mol. Chem. Neuropathol. 32, 101–121 10.1007/BF02815170 9437661

[B6] Andriollo-Sanchez M. Hininger-Favier I. Meunier N. Venneria E. O'Connor J. M. Maiani G. (2005). Age-related oxidative stress and antioxidant parameters in middle-aged and older European subjects: the ZENITH study. Eur. J. Clin. Nutr. 59(Suppl. 2), S58–S62 16254584 10.1038/sj.ejcn.1602300

[B7] Baum L. Chan I. H. Cheung S. K. Goggins W. B. Mok V. Lam L. (2010). Serum zinc is decreased in Alzheimer's disease and serum arsenic correlates positively with cognitive ability. Biometals 23, 173–179 10.1007/s10534-009-9277-5 19911117

[B8] Besser L. Chorin E. Sekler I. Silverman W. F. Atkin S. Russell J. T. (2009). Synaptically released zinc triggers metabotropic signaling via a zinc-sensing receptor in the hippocampus. J. Neurosci. 29, 2890–2901 10.1523/JNEUROSCI.5093-08.2009 19261885 PMC3175799

[B9] Beyer N. Coulson D. T. Heggarty S. Ravid R. Hellemans J. Irvine G. B. (2012). Zinc transporter mRNA levels in Alzheimer's disease postmortem brain. J. Alzheimers Dis. 29, 863–873 10.3233/JAD-2012-112105 22349685

[B10] Beyer N. Coulson D. T. Heggarty S. Ravid R. Irvine G. B. Hellemans J. (2009). ZnT3 mRNA levels are reduced in Alzheimer's disease post-mortem brain. Mol. Neurodegener. 4:53 10.1186/1750-1326-4-53 20030848 PMC2806356

[B11] Bossy-Wetzel E. Talantova M. V. Lee W. D. Scholzke M. N. Harrop A. Mathews E. (2004). Crosstalk between nitric oxide and zinc pathways to neuronal cell death involving mitochondrial dysfunction and p38-activated K+ channels. Neuron 41, 351–365 10.1016/S0896-6273(04)00015-7 14766175

[B12] Brewer G. J. Kanzer S. H. Zimmerman E. A. Molho E. S. Celmins D. F. Heckman S. M. (2010). Subclinical zinc deficiency in Alzheimer's disease and Parkinson's disease. Am. J. Alzheimers. Dis. Other Demen. 25, 572–575 10.1177/1533317510382283 20841345 PMC10845304

[B13] Burnet F. M. (1981). A possible role of zinc in the pathology of dementia. Lancet 1, 186–188 10.1016/S0140-6736(81)90062-3 6162062

[B14] Bush A. I. Pettingell W. H. Multhaup G. Paradis M. Vonsattel J. P. Gusella J. F. (1994a). Rapid induction of Alzheimer A beta amyloid formation by zinc. Science 265, 1464–1467 10.1126/science.8073293 8073293

[B15] Bush A. I. Pettingell W. H. Jr. Paradis M. D. Tanzi R. E. (1994b). Modulation of A beta adhesiveness and secretase site cleavage by zinc. J. Biol. Chem. 269, 12152–12158 8163520

[B16] Butterfield D. A. Drake J. Pocernich C. Castegna A. (2001). Evidence of oxidative damage in Alzheimer's disease brain: central role for amyloid beta-peptide. Trends Mol. Med. 7, 548–554 10.1016/S1471-4914(01)02173-6 11733217

[B17] Choi D. W. Yokoyama M. Koh J. (1988). Zinc neurotoxicity in cortical cell culture. Neuroscience 24, 67–79 10.1016/0306-4522(88)90312-0 3368058

[B18] Cichy A. Sowa-Kucma M. Legutko B. Pomierny-Chamiolo L. Siwek A. Piotrowska A. (2009). Zinc-induced adaptive changes in NMDA/glutamatergic and serotonergic receptors. Pharmacol. Rep. 61, 1184–1191 20081255 10.1016/s1734-1140(09)70182-3

[B19] Cieslik K. Klenk-Majewska B. Danilczuk Z. Wrobel A. Lupina T. Ossowska G. (2007). Influence of zinc supplementation on imipramine effect in a chronic unpredictable stress (CUS) model in rats. Pharmacol. Rep. 59, 46–52 17377205

[B20] Corona C. Frazzini V. Silvestri E. Lattanzio R. La S. R. Piantelli M. (2011). Effects of dietary supplementation of carnosine on mitochondrial dysfunction, amyloid pathology, and cognitive deficits in 3xTg-AD mice. PLoS ONE 6:e17971 10.1371/journal.pone.0017971 21423579 PMC3058055

[B21] Corona C. Masciopinto F. Silvestri E. Viscovo A. D. Lattanzio R. Sorda R. L. (2010). Dietary zinc supplementation of 3xTg-AD mice increases BDNF levels and prevents cognitive deficits as well as mitochondrial dysfunction. Cell Death Dis. 1, e91 10.1038/cddis.2010.73 21368864 PMC3035902

[B22] Corrigan F. M. Reynolds G. P. Ward N. I. (1993). Hippocampal tin, aluminum and zinc in Alzheimer's disease. Biometals 6, 149–154 10.1007/BF00205853 8400761

[B23] Cote A. Chiasson M. Peralta M. R. III. Lafortune K. Pellegrini L. Toth K. (2005). Cell type-specific action of seizure-induced intracellular zinc accumulation in the rat hippocampus. J. Physiol. 566, 821–837 10.1113/jphysiol.2005.089458 15919712 PMC1464793

[B24] Cousins R. J. Liuzzi J. P. Lichten L. A. (2006). Mammalian zinc transport, trafficking, and signals. J. Biol. Chem. 281, 24085–24089 10.1074/jbc.R600011200 16793761

[B25] Cuajungco M. P. Lees G. J. (1997). Zinc and Alzheimer's disease: is there a direct link? Brain Res. Brain Res. Rev. 23, 219–236 10.1016/S0165-0173(97)00002-7 9164672

[B26] Cunha M. P. Machado D. G. Bettio L. E. Capra J. C. Rodrigues A. L. (2008). Interaction of zinc with antidepressants in the tail suspension test. Prog. Neuropsychopharmacol. Biol. Psychiatry 32, 1913–1920 10.1016/j.pnpbp.2008.09.006 18824054

[B27] Danscher G. Jensen K. B. Frederickson C. J. Kemp K. Andreasen A. Juhl S. (1997). Increased amount of zinc in the hippocampus and amygdala of Alzheimer's diseased brains: a proton-induced X-ray emission spectroscopic analysis of cryostat sections from autopsy material. J. Neurosci. Methods 76, 53–59 10.1016/S0165-0270(97)00079-4 9334939

[B28] Daviglus M. L. Bell C. C. Berrettini W. Bowen P. E. Connolly E. S. Jr. Cox N. J. (2010). National institutes of health state-of-the-science conference statement: preventing alzheimer disease and cognitive decline. Ann. Intern. Med. 153, 176–181 10.7326/0003-4819-153-3-201008030-00260 20547888

[B29] DiGirolamo A. M. Ramirez-Zea M. Wang M. Flores-Ayala R. Martorell R. Neufeld L. M. (2010). Randomized trial of the effect of zinc supplementation on the mental health of school-age children in Guatemala. Am. J. Clin. Nutr. 92, 1241–1250 10.3945/ajcn.2010.29686 20881069 PMC2954453

[B30] Dong J. Atwood C. S. Anderson V. E. Siedlak S. L. Smith M. A. Perry G. (2003). Metal binding and oxidation of amyloid-beta within isolated senile plaque cores: Raman microscopic evidence. Biochemistry 42, 2768–2773 10.1021/bi0272151 12627941

[B31] Donnelly P. S. Caragounis A. Du T. Laughton K. M. Volitakis I. Cherny R. A. (2008). Selective intracellular release of copper and zinc ions from bis(thiosemicarbazonato) complexes reduces levels of Alzheimer disease amyloid-beta peptide. J. Biol. Chem. 283, 4568–4577 10.1074/jbc.M705957200 18086681

[B32] Erickson J. C. Sewell A. K. Jensen L. T. Winge D. R. Palmiter R. D. (1994). Enhanced neurotrophic activity in Alzheimer's disease cortex is not associated with down-regulation of metallothionein-III (GIF). Brain Res. 649, 297–304 10.1016/0006-8993(94)91076-6 7953645

[B33] Faux N. G. Ritchie C. W. Gunn A. Rembach A. Tsatsanis A. Bedo J. (2010). PBT2 rapidly improves cognition in Alzheimer's Disease: additional phase II analyses. J. Alzheimers Dis. 20, 509–516 20164561 10.3233/JAD-2010-1390

[B34] Flinn J. M. Hunter D. Linkous D. H. Lanzirotti A. Smith L. N. Brightwell J. (2005). Enhanced zinc consumption causes memory deficits and increased brain levels of zinc. Physiol. Behav. 83, 793–803 10.1016/j.physbeh.2004.10.009 15639165

[B35] Franco J. L. Posser T. Brocardo P. S. Trevisan R. Uliano-Silva M. Gabilan N. H. (2008). Involvement of glutathione, ERK1/2 phosphorylation and BDNF expression in the antidepressant-like effect of zinc in rats. Behav. Brain Res. 188, 316–323 10.1016/j.bbr.2007.11.012 18191237

[B36] Frederickson C. J. Suh S. W. Silva D. Frederickson C. J. Thompson R. B. (2000). Importance of zinc in the central nervous system: the zinc-containing neuron. J. Nutr. 130, 1471S–1483S 10801962 10.1093/jn/130.5.1471S

[B37a] Gao H. L. Xu H. Xin N. Zheng W. Chi Z. H. Wang Z. Y. (2011). Disruption of the CaMKII/CREB signaling is associated with zinc deficiency-induced learning and memory impairments. Neurotox. Res. 19, 584–591 10.1007/s12640-010-9206-y 20593259

[B37] Gao H. L. Zheng W. Xin N. Chi Z. H. Wang Z. Y. Chen J. (2009). Zinc deficiency reduces neurogenesis accompanied by neuronal apoptosis through caspase-dependent and -independent signaling pathways. Neurotox. Res. 16, 416–425 10.1007/s12640-009-9072-7 19548052

[B38] Gonzalez C. Martin T. Cacho J. Brenas M. T. Arroyo T. Garcia-Berrocal B. (1999). Serum zinc, copper, insulin and lipids in Alzheimer's disease epsilon 4 apolipoprotein E allele carriers. Eur. J. Clin. Invest. 29, 637–642 10.1046/j.1365-2362.1999.00471.x 10411671

[B39] Greenough M. A. Volitakis I. Li Q. X. Laughton K. Evin G. Ho M. (2011). Presenilins promote the cellular uptake of copper and zinc and maintain copper chaperone of SOD1-dependent copper/zinc superoxide dismutase activity. J. Biol. Chem. 286, 9776–9786 10.1074/jbc.M110.163964 21239495 PMC3058959

[B40] Grilli M. Goffi F. Memo M. Spano P. (1996). Interleukin-1beta and glutamate activate the NF-kappaB/Rel binding site from the regulatory region of the amyloid precursor protein gene in primary neuronal cultures. J. Biol. Chem. 271, 15002–15007 10.1074/jbc.271.25.15002 8663145

[B41] Haase H. Rink L. (2009). Functional significance of zinc-related signaling pathways in immune cells. Annu. Rev. Nutr. 29, 133–152 10.1146/annurev-nutr-080508-141119 19400701

[B42] Haines A. Iliffe S. Morgan P. Dormandy T. Wood B. (1991). Serum aluminium and zinc and other variables in patients with and without cognitive impairment in the community. Clin. Chim. Acta 198, 261–266 10.1016/0009-8981(91)90360-O 1889125

[B43] Hasan R. Rink L. Haase H. (2012). Zinc signals in neutrophil granulocytes are required for the formation of neutrophil extracellular traps. Innate Immun. 19, 253–264 10.1177/1753425912458815 23008348

[B44] Hershey C. O. Hershey L. A. Varnes A. Vibhakar S. D. Lavin P. Strain W. H. (1983). Cerebrospinal fluid trace element content in dementia: clinical, radiologic, and pathologic correlations. Neurology 33, 1350–1353 10.1212/WNL.33.10.1350 6684234

[B45] Huang L. Tepaamorndech S. (2013). The SLC30 family of zinc transporters - a review of current understanding of their biological and pathophysiological roles. Mol. Aspects Med. 34, 548–560 10.1016/j.mam.2012.05.008 23506888

[B46] Huang Y. Z. Pan E. Xiong Z. Q. McNamara J. O. (2008). Zinc-mediated transactivation of TrkB potentiates the hippocampal mossy fiber-CA3 pyramid synapse. Neuron 57, 546–558 10.1016/j.neuron.2007.11.026 18304484

[B47] Hwang J. J. Park M. H. Choi S. Y. Koh J. Y. (2005). Activation of the Trk signaling pathway by extracellular zinc. Role of metalloproteinases. J. Biol. Chem. 280, 11995–12001 10.1074/jbc.M403172200 15659400

[B48] Irmisch G. Schlaefke D. Richter J. (2010). Zinc and fatty acids in depression. Neurochem. Res. 35, 1376–1383 10.1007/s11064-010-0194-3 20524151

[B49] Jeandel C. Nicolas M. B. Dubois F. Nabet-Belleville F. Penin F. Cuny G. (1989). Lipid peroxidation and free radical scavengers in Alzheimer's disease. Gerontology 35, 275–282 10.1159/000213037 2630382

[B50] Jomova K. Vondrakova D. Lawson M. Valko M. (2010). Metals, oxidative stress and neurodegenerative disorders. Mol. Cell Biochem. 345, 91–104 10.1007/s11010-010-0563-x 20730621

[B51] Kahmann L. Uciechowski P. Warmuth S. Plumakers B. Gressner A. M. Malavolta M. (2008). Zinc supplementation in the elderly reduces spontaneous inflammatory cytokine release and restores T cell functions. Rejuvenation Res. 11, 227–237 10.1089/rej.2007.0613 18279033

[B52] Katzman R. Aronson M. Fuld P. Kawas C. Brown T. Morgenstern H. (1989). Development of dementing illnesses in an 80-year-old volunteer cohort. Ann. Neurol. 25, 317–324 10.1002/ana.410250402 2712531

[B53] Keller K. A. Grider A. Coffield J. A. (2001). Age-dependent influence of dietary zinc restriction on short-term memory in male rats. Physiol. Behav. 72, 339–348 10.1016/S0031-9384(00)00421-2 11274675

[B54] Kelly E. J. Quaife C. J. Froelick G. J. Palmiter R. D. (1996). Metallothionein I and II protect against zinc deficiency and zinc toxicity in mice. J. Nutr. 126, 1782–1790 8683339 10.1093/jn/126.7.1782

[B55] Krezel A. Hao Q. Maret W. (2007). The zinc/thiolate redox biochemistry of metallothionein and the control of zinc ion fluctuations in cell signaling. Arch. Biochem. Biophys. 463, 188–200 10.1016/j.abb.2007.02.017 17391643

[B56] Kroczka B. Branski P. Palucha A. Pilc A. Nowak G. (2001). Antidepressant-like properties of zinc in rodent forced swim test. Brain Res. Bull. 55, 297–300 10.1016/S0361-9230(01)00473-7 11470330

[B57] Kroczka B. Zieba A. Dudek D. Pilc A. Nowak G. (2000). Zinc exhibits an antidepressant-like effect in the forced swimming test in mice. Pol. J. Pharmacol. 52, 403–406 11334234

[B58] Lang M. Wang L. Fan Q. Xiao G. Wang X. Zhong Y. (2012). Genetic inhibition of solute-linked carrier 39 family transporter 1 ameliorates abeta pathology in a Drosophila model of Alzheimer's disease. PLoS Genet. 8:e1002683 10.1371/journal.pgen.1002683 22570624 PMC3343105

[B59] Lannfelt L. Blennow K. Zetterberg H. Batsman S. Ames D. Harrison J. (2008). Safety, efficacy, and biomarker findings of PBT2 in targeting Abeta as a modifying therapy for Alzheimer's disease: a phase IIa, double-blind, randomised, placebo-controlled trial. Lancet Neurol. 7, 779–786 10.1016/S1474-4422(08)70167-4 18672400

[B60] Lee J. Kim C. H. Kim D. G. Ahn Y. S. (2009). Zinc inhibits amyloid beta production from Alzheimer's amyloid precursor protein in SH-SY5Y Cells. Korean J. Physiol. Pharmacol. 13, 195–200 10.4196/kjpp.2009.13.3.195 19885037 PMC2766728

[B61] Lee J. Y. Cole T. B. Palmiter R. D. Suh S. W. Koh J. Y. (2002). Contribution by synaptic zinc to the gender-disparate plaque formation in human Swedish mutant APP transgenic mice. Proc. Natl. Acad. Sci. U.S.A. 99, 7705–7710 10.1073/pnas.092034699 12032347 PMC124328

[B62] Lee J. Y. Friedman J. E. Angel I. Kozak A. Koh J. Y. (2004). The lipophilic metal chelator DP-109 reduces amyloid pathology in brains of human beta-amyloid precursor protein transgenic mice. Neurobiol. Aging 25, 1315–1321 10.1016/j.neurobiolaging.2004.01.005 15465629

[B63] Lichten L. A. Cousins R. J. (2009). Mammalian zinc transporters: nutritional and physiologic regulation. Annu. Rev. Nutr. 29, 153–176 10.1146/annurev-nutr-033009-083312 19400752

[B64] Ling Y. Morgan K. Kalsheker N. (2003). Amyloid precursor protein (APP) and the biology of proteolytic processing: relevance to Alzheimer's disease. Int. J. Biochem. Cell Biol. 35, 1505–1535 10.1016/S1357-2725(03)00133-X 12824062

[B65] Liuzzi J. P. Cousins R. J. (2004). Mammalian zinc transporters. Annu. Rev. Nutr. 24, 151–172 10.1146/annurev.nutr.24.012003.132402 15189117

[B66] Lovell M. A. (2009). A potential role for alterations of zinc and zinc transport proteins in the progression of Alzheimer's disease. J. Alzheimers Dis. 16, 471–483 19276540 10.3233/JAD-2009-0992 PMC2881701

[B67] Lovell M. A. Robertson J. D. Teesdale W. J. Campbell J. L. Markesbery W. R. (1998). Copper, iron and zinc in Alzheimer's disease senile plaques. J. Neurol. Sci. 158, 47–52 10.1016/S0022-510X(98)00092-6 9667777

[B68] Lovell M. A. Smith J. L. Markesbery W. R. (2006). Elevated zinc transporter-6 in mild cognitive impairment, Alzheimer disease, and pick disease. J. Neuropathol. Exp. Neurol. 65, 489–498 10.1097/01.jnen.0000229237.98124.91 16772872

[B69] Lovell M. A. Smith J. L. Xiong S. Markesbery W. R. (2005). Alterations in zinc transporter protein-1 (ZnT-1) in the brain of subjects with mild cognitive impairment, early, and late-stage Alzheimer's disease. Neurotox. Res. 7, 265–271 10.1007/BF03033884 16179263

[B70] Lyubartseva G. Lovell M. A. (2012). A potential role for zinc alterations in the pathogenesis of Alzheimer's disease. Biofactors 38, 98–106 10.1002/biof.199 22447723 PMC3635097

[B71] Lyubartseva G. Smith J. L. Markesbery W. R. Lovell M. A. (2010). Alterations of zinc transporter proteins ZnT-1, ZnT-4 and ZnT-6 in preclinical Alzheimer's disease brain. Brain Pathol. 20, 343–350 10.1111/j.1750-3639.2009.00283.x 19371353 PMC3175637

[B72] Maes M. Bosmans E. De Jongh R. Kenis G. Vandoolaeghe E. Neels H. (1997). Increased serum IL-6 and IL-1 receptor antagonist concentrations in major depression and treatment resistant depression. Cytokine 9, 853–858 10.1006/cyto.1997.0238 9367546

[B73] Maes M. D'Haese P. C. Scharpe S. D'Hondt P. Cosyns P. De Broe M. E. (1994). Hypozincemia in depression. J. Affect. Disord. 31, 135–140 10.1016/0165-0327(94)90117-1 8071476

[B74] Maes M. Yirmyia R. Noraberg J. Brene S. Hibbeln J. Perini G. (2009). The inflammatory and neurodegenerative (IandND) hypothesis of depression: leads for future research and new drug developments in depression. Metab. Brain Dis. 24, 27–53 10.1007/s11011-008-9118-1 19085093

[B75] Masters B. A. Quaife C. J. Erickson J. C. Kelly E. J. Froelick G. J. Zambrowicz B. P. (1994). Metallothionein III is expressed in neurons that sequester zinc in synaptic vesicles. J. Neurosci. 14, 5844–5857 7931547 10.1523/JNEUROSCI.14-10-05844.1994 PMC6577000

[B76] Masters C. L. Simms G. Weinman N. A. Multhaup G. McDonald B. L. Beyreuther K. (1985). Amyloid plaque core protein in Alzheimer disease and Down syndrome. Proc. Natl. Acad. Sci. U.S.A. 82, 4245–4249 10.1073/pnas.82.12.4245 3159021 PMC397973

[B77] McLoughlin I. J. Hodge J. S. (1990). Zinc in depressive disorder. Acta Psychiatr. Scand. 82, 451–453 10.1111/j.1600-0447.1990.tb03077.x 2291414

[B78] Miller L. M. Wang Q. Telivala T. P. Smith R. J. Lanzirotti A. Miklossy J. (2006). Synchrotron-based infrared and X-ray imaging shows focalized accumulation of Cu and Zn co-localized with beta-amyloid deposits in Alzheimer's disease. J. Struct. Biol. 155, 30–37 10.1016/j.jsb.2005.09.004 16325427

[B79] Mlyniec K. Budziszewska B. Reczynski W. Sowa-Kucma M. Nowak G. (2013). The role of the GPR39 receptor in zinc deficient-animal model of depression. Behav. Brain Res. 238, 30–35 10.1016/j.bbr.2012.10.020 23089648

[B80] Mlyniec K. Davies C. L. Budziszewska B. Opoka W. Reczynski W. Sowa-Kucma M. (2012). Time course of zinc deprivation-induced alterations of mice behavior in the forced swim test. Pharmacol. Rep. 64, 567–575 22814010 10.1016/s1734-1140(12)70852-6

[B81] Mlyniec K. Nowak G. (2012). Zinc deficiency induces behavioral alterations in the tail suspension test in mice. Effect of antidepressants. Pharmacol. Rep. 64, 249–255 22661173 10.1016/s1734-1140(12)70762-4

[B82] Mlyniec K. Nowak G. (2013). GPR39 up-regulation after selective antidepressants. Neurochem. Int. 62, 936–939 10.1016/j.neuint.2013.02.024 23474197

[B83] Mocchegiani E. Bertoni-Freddari C. Marcellini F. Malavolta M. (2005). Brain, aging and neurodegeneration: role of zinc ion availability. Prog. Neurobiol. 75, 367–390 10.1016/j.pneurobio.2005.04.005 15927345

[B84] Mocchegiani E. Burkle A. Fulop T. (2008). Zinc and ageing (ZINCAGE Project). Exp. Gerontol. 43, 361–362 10.1016/j.exger.2008.03.009 18417310

[B85] Mocchegiani E. Costarelli L. Giacconi R. Piacenza F. Basso A. Malavolta M. (2011). Zinc, metallothioneins and immunosenescence: effect of zinc supply as nutrigenomic approach. Biogerontology 12, 455–465 10.1007/s10522-011-9337-4 21503725

[B86] Mocchegiani E. Malavolta M. Costarelli L. Giacconi R. Cipriano C. Piacenza F. (2010). Zinc, metallothioneins and immunosenescence. Proc. Nutr. Soc. 69, 290–299 10.1017/S0029665110001862 20579408

[B87] Molina J. A. Jimenez-Jimenez F. J. Aguilar M. V. Meseguer I. Mateos-Vega C. J. Gonzalez-Munoz M. J. (1998). Cerebrospinal fluid levels of transition metals in patients with Alzheimer's disease. J. Neural Transm. 105, 479–488 10.1007/s007020050071 9720975

[B88] Narang R. L. Gupta K. R. Narang A. P. Singh R. (1991). Levels of copper and zinc in depression. Indian J. Physiol. Pharmacol. 35, 272–274 1812105

[B89] Nguyen P. H. Grajeda R. Melgar P. Marcinkevage J. DiGirolamo A. M. Flores R. (2009). Micronutrient supplementation may reduce symptoms of depression in Guatemalan women. Arch. Latinoam. Nutr. 59, 278–286 19886513

[B90] Noh K. M. Koh J. Y. (2000). Induction and activation by zinc of NADPH oxidase in cultured cortical neurons and astrocytes. J. Neurosci. 20, RC111 11090611 10.1523/JNEUROSCI.20-23-j0001.2000 PMC6773049

[B91] Nowak G. Siwek M. Dudek D. Zieba A. Pilc A. (2003a). Effect of zinc supplementation on antidepressant therapy in unipolar depression: a preliminary placebo-controlled study. Pol. J. Pharmacol. 55, 1143–1147 14730113

[B92] Nowak G. Szewczyk B. Wieronska J. M. Branski P. Palucha A. Pilc A. (2003b). Antidepressant-like effects of acute and chronic treatment with zinc in forced swim test and olfactory bulbectomy model in rats. Brain Res. Bull. 61, 159–164 10.1016/S0361-9230(03)00104-7 12832002

[B93] Nowak G. Zieba A. Dudek D. Krosniak M. Szymaczek M. Schlegel-Zawadzka M. (1999). Serum trace elements in animal models and human depression. Part I. Zinc. Hum. Psychopharmacol. Clin. Exp. 14, 83–86 10.1002/hup.231 12404616

[B94] Panayi A. E. Spyrou N. M. Iversen B. S. White M. A. Part P. (2002). Determination of cadmium and zinc in Alzheimer's brain tissue using inductively coupled plasma mass spectrometry. J. Neurol. Sci. 195, 1–10 10.1016/S0022-510X(01)00672-4 11867068

[B95] Paoletti P. Vergnano A. M. Barbour B. Casado M. (2009). Zinc at glutamatergic synapses. Neuroscience 158, 126–136 10.1016/j.neuroscience.2008.01.061 18353558

[B96] Perry D. K. Smyth M. J. Stennicke H. R. Salvesen G. S. Duriez P. Poirier G. G. (1997). Zinc is a potent inhibitor of the apoptotic protease, caspase-3. A novel target for zinc in the inhibition of apoptosis. J. Biol. Chem. 272, 18530–18533 10.1074/jbc.272.30.18530 9228015

[B97] Piechal A. Blecharz-Klin K. Pyrzanowska J. Widy-Tyszkiewicz E. (2012). Maternal zinc supplementation improves spatial memory in rat pups. Biol. Trace Elem. Res. 147, 299–308 10.1007/s12011-012-9323-y 22249889 PMC3362702

[B98] Plum L. M. Rink L. Haase H. (2010). The essential toxin: impact of zinc on human health. Int. J. Environ. Res. Public Health 7, 1342–1365 10.3390/ijerph7041342 20617034 PMC2872358

[B99] Quaife C. J. Findley S. D. Erickson J. C. Froelick G. J. Kelly E. J. Zambrowicz B. P. (1994). Induction of a new metallothionein isoform (MT-IV) occurs during differentiation of stratified squamous epithelia. Biochemistry 33, 7250–7259 10.1021/bi00189a029 8003488

[B100] Railey A. M. Micheli T. L. Wanschura P. B. Flinn J. M. (2010). Alterations in fear response and spatial memory in pre- and post-natal zinc supplemented rats: remediation by copper. Physiol. Behav. 100, 95–100 10.1016/j.physbeh.2010.01.040 20159028

[B101] Ranjbar E. Shams J. Sabetkasaei M. Shirazi M. Rashidkhani B. Mostafavi A. (2013). Effects of zinc supplementation on efficacy of antidepressant therapy, inflammatory cytokines, and brain-derived neurotrophic factor in patients with major depression. Nutr. Neurosci. [Epub ahead of print]. 10.1179/1476830513Y.0000000066 23602205

[B102] Religa D. Strozyk D. Cherny R. A. Volitakis I. Haroutunian V. Winblad B. (2006). Elevated cortical zinc in Alzheimer disease. Neurology 67, 69–75 10.1212/01.wnl.0000223644.08653.b5 16832080

[B103] Roozbeh J. Sharifian M. Ghanizadeh A. Sahraian A. Sagheb M. M. Shabani S. (2011). Association of zinc deficiency and depression in the patients with end-stage renal disease on hemodialysis. J. Ren. Nutr. 21, 184–187 10.1053/j.jrn.2010.05.015 21093288

[B104] Rosa A. O. Lin J. Calixto J. B. Santos A. R. Rodrigues A. L. (2003). Involvement of NMDA receptors and L-arginine-nitric oxide pathway in the antidepressant-like effects of zinc in mice. Behav. Brain Res. 144, 87–93 10.1016/S0166-4328(03)00069-X 12946598

[B105] Roy A. Evers S. E. Avison W. R. Campbell M. K. (2010). Higher zinc intake buffers the impact of stress on depressive symptoms in pregnancy. Nutr. Res. 30, 695–704 10.1016/j.nutres.2010.09.011 21056285

[B106] Rulon L. L. Robertson J. D. Lovell M. A. Deibel M. A. Ehmann W. D. Markesber W. R. (2000). Serum zinc levels and Alzheimer's disease. Biol. Trace Elem. Res. 75, 79–85 10.1385/BTER:75:1-3:79 11051598

[B107] Sahu R. N. Pandey R. S. Subhash M. N. Arya B. Y. Padmashree T. S. Srinivas K. N. (1988). CSF zinc in Alzheimer's type dementia. Biol. Psychiatry 24, 480–482 10.1016/0006-3223(88)90190-4 3408766

[B108] Samudralwar D. L. Diprete C. C. Ni B. F. Ehmann W. D. Markesbery W. R. (1995). Elemental imbalances in the olfactory pathway in Alzheimer's disease. J. Neurol. Sci. 130, 139–145 10.1016/0022-510X(95)00018-W 8586977

[B109] Sandstead H. H. (2012). Subclinical zinc deficiency impairs human brain function. J. Trace Elem. Med. Biol. 26, 70–73 10.1016/j.jtemb.2012.04.018 22673824

[B110] Sato M. Kondoh M. (2002). Recent studies on metallothionein: protection against toxicity of heavy metals and oxygen free radicals. Tohoku J. Exp. Med. 196, 9–22 10.1620/tjem.196.9 12498322

[B111] Sawada T. Yokoi K. (2010). Effect of zinc supplementation on mood states in young women: a pilot study. Eur. J. Clin. Nutr. 64, 331–333 10.1038/ejcn.2009.158 20087376

[B112] Sensi S. L. Paoletti P. Koh J. Y. Aizenman E. Bush A. I. Hershfinkel M. (2011). The neurophysiology and pathology of brain zinc. J. Neurosci. 31, 16076–16085 10.1523/JNEUROSCI.3454-11.2011 22072659 PMC3223736

[B113] Sensi S. L. Rapposelli I. G. Frazzini V. Mascetra N. (2008). Altered oxidant-mediated intraneuronal zinc mobilization in a triple transgenic mouse model of Alzheimer's disease. Exp. Gerontol. 43, 488–492 10.1016/j.exger.2007.10.018 18068923

[B114] Sensi S. L. Ton-That D. Sullivan P. G. Jonas E. A. Gee K. R. Kaczmarek L. K. (2003). Modulation of mitochondrial function by endogenous Zn2+ pools. Proc. Natl. Acad. Sci. U.S.A. 100, 6157–6162 10.1073/pnas.1031598100 12724524 PMC156342

[B115] Shore D. Henkin R. I. Nelson N. R. Agarwal R. P. Wyatt R. J. (1984). Hair and serum copper, zinc, calcium, and magnesium concentrations in Alzheimer-type dementia. J. Am. Geriatr. Soc. 32, 892–895 6512128 10.1111/j.1532-5415.1984.tb00889.x

[B116] Siwek M. Dudek D. Paul I. A. Sowa-Kucma M. Zieba A. Popik P. (2009). Zinc supplementation augments efficacy of imipramine in treatment resistant patients: a double blind, placebo-controlled study. J. Affect. Disord. 118, 187–195 10.1016/j.jad.2009.02.014 19278731

[B117] Siwek M. Dudek D. Schlegel-Zawadzka M. Morawska A. Piekoszewski W. Opoka W. (2010). Serum zinc level in depressed patients during zinc supplementation of imipramine treatment. J. Affect. Disord. 126, 447–452 10.1016/j.jad.2010.04.024 20493532

[B118] Small D. H. Cappai R. (2006). Alois Alzheimer and Alzheimer's disease: a centennial perspective. J. Neurochem. 99, 708–710 10.1111/j.1471-4159.2006.04212.x 17076655

[B119] Smart T. G. Hosie A. M. Miller P. S. (2004). Zn2+ ions: modulators of excitatory and inhibitory synaptic activity. Neuroscientist 10, 432–442 10.1177/1073858404263463 15359010

[B120] Smith J. L. Xiong S. Markesbery W. R. Lovell M. A. (2006). Altered expression of zinc transporters-4 and -6 in mild cognitive impairment, early and late Alzheimer's disease brain. Neuroscience 140, 879–888 10.1016/j.neuroscience.2006.02.049 16580781

[B121] Sowa-Kucma M. Legutko B. Szewczyk B. Novak K. Znojek P. Poleszak E. (2008). Antidepressant-like activity of zinc: further behavioral and molecular evidence. J. Neural Transm. 115, 1621–1628 10.1007/s00702-008-0115-7 18766297

[B122] Suh S. W. Jensen K. B. Jensen M. S. Silva D. S. Kesslak P. J. Danscher G. (2000). Histochemically-reactive zinc in amyloid plaques, angiopathy, and degenerating neurons of Alzheimer's diseased brains. Brain Res. 852, 274–278 10.1016/S0006-8993(99)02096-X 10678753

[B123] Suh S. W. Won S. J. Hamby A. M. Yoo B. H. Fan Y. Sheline C. T. (2009). Decreased brain zinc availability reduces hippocampal neurogenesis in mice and rats. J. Cereb. Blood Flow Metab. 29, 1579–1588 10.1038/jcbfm.2009.80 19536073

[B124] Szewczyk B. Branski P. Wieronska J. M. Palucha A. Pilc A. Nowak G. (2002). Interaction of zinc with antidepressants in the forced swimming test in mice. Pol. J. Pharmacol. 54, 681–685 12866724

[B125] Szewczyk B. Poleszak E. Sowa-Kucma M. Wrobel A. Slotwinski S. Listos J. (2010). The involvement of NMDA and AMPA receptors in the mechanism of antidepressant-like action of zinc in the forced swim test. Amino Acids 39, 205–217 10.1007/s00726-009-0412-y 19956994

[B126] Szewczyk B. Poleszak E. Wlaz P. Wrobel A. Blicharska E. Cichy A. (2009). The involvement of serotonergic system in the antidepressant effect of zinc in the forced swim test. Prog. Neuropsychopharmacol. Biol. Psychiatry 33, 323–329 10.1016/j.pnpbp.2008.12.011 19150479

[B127] Takeda A. (2000). Movement of zinc and its functional significance in the brain. Brain Res. Brain Res. Rev. 34, 137–148 10.1016/S0165-0173(00)00044-8 11113504

[B128] Takeda A. Tamano H. Tochigi M. Oku N. (2005). Zinc homeostasis in the hippocampus of zinc-deficient young adult rats. Neurochem. Int. 46, 221–225 10.1016/j.neuint.2004.10.003 15670638

[B129] Tamano H. Kan F. Kawamura M. Oku N. Takeda A. (2009). Behavior in the forced swim test and neurochemical changes in the hippocampus in young rats after 2-week zinc deprivation. Neurochem. Int. 55, 536–541 10.1016/j.neuint.2009.05.011 19463882

[B130] Tassabehji N. M. Corniola R. S. Alshingiti A. Levenson C. W. (2008). Zinc deficiency induces depression-like symptoms in adult rats. Physiol Behav. 95, 365–369 10.1016/j.physbeh.2008.06.017 18655800

[B131] Thompson C. M. Markesbery W. R. Ehmann W. D. Mao Y. X. Vance D. E. (1988). Regional brain trace-element studies in Alzheimer's disease. Neurotoxicology 9, 1–7 3393299

[B132] Trombley P. Q. Horning M. S. Blakemore L. J. (1998). Carnosine modulates zinc and copper effects on amino acid receptors and synaptic transmission. Neuroreport 9, 3503–3507 10.1097/00001756-199810260-00031 9855307

[B133] Tsuji S. Kobayashi H. Uchida Y. Ihara Y. Miyatake T. (1992). Molecular cloning of human growth inhibitory factor cDNA and its down-regulation in Alzheimer's disease. EMBO J. 11, 4843–4850 1464312 10.1002/j.1460-2075.1992.tb05590.x PMC556960

[B134] Uchida Y. Takio K. Titani K. Ihara Y. Tomonaga M. (1991). The growth inhibitory factor that is deficient in the Alzheimer's disease brain is a 68 amino acid metallothionein-like protein. Neuron 7, 337–347 10.1016/0896-6273(91)90272-2 1873033

[B135] Vural H. Demirin H. Kara Y. Eren I. Delibas N. (2010). Alterations of plasma magnesium, copper, zinc, iron and selenium concentrations and some related erythrocyte antioxidant enzyme activities in patients with Alzheimer's disease. J.Trace Elem. Med. Biol. 24, 169–173 10.1016/j.jtemb.2010.02.002 20569929

[B136] Wang T. Wang C. Y. Shan Z. Y. Teng W. P. Wang Z. Y. (2012). Clioquinol reduces zinc accumulation in neuritic plaques and inhibits the amyloidogenic pathway in AbetaPP/PS1 transgenic mouse brain. J. Alzheimers Dis. 29, 549–559 22269164 10.3233/JAD-2011-111874

[B137] Watanabe M. Tamano H. Kikuchi T. Takeda A. (2010). Susceptibility to stress in young rats after 2-week zinc deprivation. Neurochem. Int. 56, 410–416 10.1016/j.neuint.2009.11.014 19931332

[B138] Wenstrup D. Ehmann W. D. Markesbery W. R. (1990). Trace element imbalances in isolated subcellular fractions of Alzheimer's disease brains. Brain Res. 533, 125–131 10.1016/0006-8993(90)91804-P 2085723

[B139] Whittle N. Lubec G. Singewald N. (2009). Zinc deficiency induces enhanced depression-like behaviour and altered limbic activation reversed by antidepressant treatment in mice. Amino Acids 36, 147–158 10.1007/s00726-008-0195-6 18975044

[B140] Wilquet V. De S. B. (2004). Amyloid-beta precursor protein processing in neurodegeneration. Curr. Opin. Neurobiol. 14, 582–588 10.1016/j.conb.2004.08.001 15464891

[B141] Wojcik J. Dudek D. Schlegel-Zawadzka M. Grabowska M. Marcinek A. Florek E. (2006). Antepartum/postpartum depressive symptoms and serum zinc and magnesium levels. Pharmacol. Rep. 58, 571–576 16963806

[B142] Wong C. P. Magnusson K. R. Ho E. (2013). Increased inflammatory response in aged mice is associated with age-related zinc deficiency and zinc transporter dysregulation. J. Nutr. Biochem. 24, 353–359 10.1016/j.jnutbio.2012.07.005 22981370 PMC3586240

[B143] Yang Y. Jing X. P. Zhang S. P. Gu R. X. Tang F. X. Wang X. L. (2013). High dose zinc supplementation induces hippocampal zinc deficiency and memory impairment with inhibition of BDNF signaling. PLoS ONE 8:e55384 10.1371/journal.pone.0055384 23383172 PMC3561272

[B144] Yu W. H. Lukiw W. J. Bergeron C. Niznik H. B. Fraser P. E. (2001). Metallothionein III is reduced in Alzheimer's disease. Brain Res. 894, 37–45 10.1016/S0006-8993(00)03196-6 11245813

[B145] Zhang L. H. Wang X. Stoltenberg M. Danscher G. Huang L. Wang Z. Y. (2008). Abundant expression of zinc transporters in the amyloid plaques of Alzheimer's disease brain. Brain Res. Bull. 77, 55–60 10.1016/j.brainresbull.2008.03.014 18639746

[B146] Zhang L. H. Wang X. Zheng Z. H. Ren H. Stoltenberg M. Danscher G. (2010). Altered expression and distribution of zinc transporters in APP/PS1 transgenic mouse brain. Neurobiol. Aging 31, 74–87 10.1016/j.neurobiolaging.2008.02.018 18378045

